# Supplementary material for: Collaborative intelligence in AI: Evaluating the performance of a council of AIs on the USMLE
Source: PLOS Digit Health. 2025 Oct 9;4(10):e0000787. doi: 10.1371/journal.pdig.0000787 (PMC12510544; doi:10.1371/journal.pdig.0000787)
Supplement: S2 File — (ZIP) [file pdig.0000787.s002.zip › S2 Data Raw data files containing unprocessed question inputs and LLM outputs including transcripts of deliberations/STEP3_Question-49-1_Type-MC-NJ.docx]

**Facilitator** **[Round 1]:** A 35-year-old woman comes to the office for follow-up of chronic pelvic pain. The patient has had deep pelvic pain since undergoing total abdominal hysterectomy and radiation therapy for treatment of cervical cancer 5 years ago. She reports constant, severe aching pain; she rates the pain at its maximum as a 10 on a 10‐point scale. The pain worsens during intercourse. All subsequent cancer follow-up has been negative. Medical history otherwise is unremarkable. The patient has taken oxycodone for her pain for 3 years, but she now reports that the oxycodone only partially relieves her pain. She requests either an increased dose of oxycodone or a change in her prescription to controlled-release morphine, which was recently given to her by a friend. She has smoked one pack of cigarettes daily for the past 15 years. She does not drink alcoholic beverages. BMI is 18 kg/m². She appears lethargic. She is oriented to person, place, and time but speaks slowly with slurred speech. Vital signs are normal. Examination of the skin shows no abnormalities. Pupils are pinpoint and react sluggishly. Which of the following is the most appropriate next step in management? (A) Inform the patient that she must find a new physician (B) Maintain the dose of oxycodone and add naproxen (C) Order a urine toxicology screening (D) Report the patient's unauthorized drug use to the police (E) Switch oxycodone to sustained-release morphine

**AI_C:** RESTATING THE QUESTION: The query poses the question of how best to manage a 35-year-old woman's chronic pelvic pain, supposedly resulting from a total abdominal hysterectomy and radiation therapy she underwent for cervical cancer 5 years prior. Oxycodone, her previous painkillers, no longer seem as effective, and she's requesting a higher dose or a switch to controlled-release morphine. Her lethargic appearance, slow, slurred speech, pinpoint pupils, and sluggish pupil response may be indicative of opioid misuse. PLANNING: Frankly, we need to address two main issues here: the chronic pelvic pain and the potential opioid misuse. While her uncontrolled pain and desire to switch or increase medication might suggest hyperalgesia or tolerance typical with long-term opioid use, her physical signs hint at opioid misuse. Hyperalgesia is heightened sensitivity to pain, while tolerance is diminished response to the drug dose. We could hypothetically increase pain management or aid her in detoxifying and dealing with possible misuse. CLINICAL REASONING: As medicine is a patient-centered practice, our focus is always to first do no harm. As such, practising due diligence in assessing her potential misuse while concurrently addressing her incessant pain is vital. Just switching her opioid or increasing the dosage without discerning opioid misuse might aggravate a potential substance use disorder. Hence, first, we need to confirm the suspected opioid misuse. CONCLUDING: The most suitable course would be an initial urine toxicology screen to help evaluate the possible misuse. This process is quick, non-invasive, and would provide valuable insight into the patient's opioid use, helping us formulate an apt treatment plan. RESPONSE: Therefore, the most appropriate next step in management would be to Option C: Order a urine toxicology screening.

**AI_E:** RESTATING THE QUESTION: This question presents a 35-year-old woman diagnosed with chronic pelvic pain following the treatment for cervical cancer which occurred 5 years ago. The patient has been relying on oxycodone to manage her pain for the past 3 years, a drug that's progressively losing its efficacy. She is currently requesting for an increase in dosage or a switch to controlled-release morphine, a drug she acquired through unofficial means. In addition to the pain, she shows signs of lethargy and her pupils react sluggishly - symptoms which may be associated with her long-term use of opioids. With these details, we are tasked with identifying the most appropriate next step in managing her case. PLANNING: The assumptions we can identify in this case include the constant pelvic pain linked to her previous cancer treatment, the failing efficacy of oxycodone, her request for higher dosage or a switch in medication to morphine, her potential misuse of opioids evident by her unorthodox access to morphine and slow reflexes. From these assumptions, we can generate the alternate assumption that perhaps the patient is showing signs of opioid misuse or dependency. Hence, it's essential to clinically prioritize this alternate assumption in the following steps. CLINICAL REASONING: Chronic use of opioids like oxycodone can develop into opioid dependency, with a typical symptom being that the drug can no longer provide the same level of pain relief at the current dosage. As the patient attempts to maintain the pain relief effect of the opioids, they may resort to increasing their doses or finding stronger alternatives, such as morphine in this case. This patient's signs of lethargy, slow speech, and pinpoint pupils further suggest the abuse of opioids. CONCLUDING: Given these clinical presentations, it would be clinically responsible to address the indication of opioid misuse before making adjustments to her pain management regimen. RESPONSE: Therefore, option (C) 'Order a urine toxicology screening' in order to confirm the suspicion of opioid misuse would be the most appropriate next step in management of this patient.

**AI_B:** 1) RESTATING THE QUESTION: This is a case about a 35-year-old female who has chronic pelvic pain following a total abdominal hysterectomy and radiation therapy for cervical cancer 5 years ago. Her oncological prognosis appears to be promising as all subsequent cancer follow-ups have been negative. She's been on oxycodone, an opioid medication, for pain management for 3 years. However, she's now reporting insufficient relief from the medication and requests an increase in dosage or a switch to morphine, which she obtained without a prescription from a friend. This patient also has a history of chronic cigarette smoking. Physical exam findings include lethargy, slow speech, and pinpoint pupils that are sluggishly reactive—symptoms that might suggest opioid misuse or overdose. Therefore, the question posits what the most appropriate next step in her management would be. 2) PLANNING: We need to consider the assumption that she may be suffering from opioid overuse or misuse, which can be confirmed by a urine toxicology screen (C). Additionally, we might question whether she's developed a tolerance to the opioid and therefore requires a higher dose or a different type of opioid like morphine (E), or whether an alternative intervention, such as adding an anti-inflammatory medication like naproxen (B), would help manage her pain. However, given her examination findings and the fact that she illegally obtained morphine from a friend, the possibility of opioid misuse seems most prominent. 3) CLINICAL REASONING: Chronic pelvic pain following cancer treatments like hysterectomy and radiation therapy isn't unusual and may be due to radiation effects or nerve damage. While the pain is legitimate and sincere, the patient's request for increased opioids or a transition to morphine raises concerns, especially when paired with physical signs consistent with opioid intoxication (e.g., lethargy, slow/slurred speech, pinpoint pupils). This constellation suggests that she might be using opioids beyond their medically indicated use. 4) CONCLUDING: When patients exhibit signs of potential substance misuse, it's important for healthcare providers to direct efforts towards confirming suspicions and subsequently addressing the misuse rather than immediately altering the prescription regimen or dismissing the patient from the practice. Reporting the patient's actions to the police (D) isn't beneficial in terms of patient care, and dismissing the patient (A) could potentially exacerbate the problem. On the other hand, blindly escalating her opioid prescription (B and E) could further fuel her potential misuse. 5) RESPONSE: Thus, the next most appropriate step in management would be to order a urine toxicology screening (C) to confirm suspicions of opioid misuse. If the patient's urine tests positive for opioids, it will be necessary to address the misuse through means like careful monitoring, providing psychoeducation, initiating treatment for substance use disorder, or referring the patient to addiction specialists as necessary.

**AI_D:** RESTATING THE QUESTION: A 35-year-old female patient who underwent treatment for cervical cancer 5 years ago is suffering from chronic pelvic pain. She has been on oxycodone for the past 3 years but reports that it only partially manages her pain. Despite her normal vital signs and absence of physical abnormalities, she demonstrates signs of lethargy and sluggish pupil reaction. She admits to using controlled-release morphine given to her by a friend and requests an increased dose of oxycodone or a change in her prescription to morphine. We are seeking guidance on what is the most appropriate next action to take. PLANNING: At this stage, given the symptoms manifested by the patient (lethargy, slurred speech, pinpoint and sluggish pupils), it is reasonable to assume that the patient may be misusing the opioids for nonmedical reasons or has developed a dependence, which is common among long-term opioid users. Thus, it would be critical to determine the extent and nature of this potential misuse or dependence. Alternatively, it could be hypothesized that the patient may be experiencing undertreated pain that requires higher doses or alternative pain medications, although her physical examination does not clearly justify this assumption. CLINICAL REASONING: Opioids, such as oxycodone and morphine, can have serious side effects including dependence, tolerance, and potential addiction. Furthermore, opioid misuse increases the risk of overdose, which can cause myosis (pupillary constriction), lethargy, and slurred speech, much like the signs observed in our patient. Considering these potential risks when chronically administering opioids, it's crucial to regularly reassess patients' pain and functionality, as well as the presence of adverse effects or aberrant behavior. The latter may suggest a substance use disorder, with the patient's unapproved use of morphine from a friend raising considerable red flags. Continuation of the current therapy or switching to a different opioid without addressing these concerns and behaviors risks the patient's health and violates ethical duty. CONCLUDING: Given these considerations, it would be unwise to increase the dose or switch the opioids without assessing whether a disorder exists. Aberrant drug behaviors should be addressed and explored before making changes to the opioid regimen. Therefore, the primary course of action should be an evaluation for a potential opioid use disorder. RESPONSE: The most appropriate next step in managing this patient would be to perform a urine toxicology screen (Option C). This approach would help us to identify the presence any illegal substances or validate the correct usage of prescribed medication, ensuring the safety and well-being of the patient moving forward. It may be warranted to recommend her for psychotherapy or refer her to a pain management or addiction specialist based on the outcomes of the screening and evaluation.

**AI_A:** RESTATING THE QUESTION: This query presents a situation of a 35-year-old woman suffering from chronic pelvic pain since her total abdominal hysterectomy and radiation therapy for cervical cancer. Her kind of pain is described as severe, and it escalates during sexual intercourse. The cancer follow-ups have been negative, hence, cancer-related pain could be ruled out. She has been taking oxycodone for three years without significant improvement. The patient's history also mentions the unauthorized use of controlled-release morphine given to her by a friend. Besides, she manifests pinpoint and sluggishly reacting pupils, lethargic appearance, slow speaking rate, and slurred speech. These symptoms, combined with her regular smoking habits and lowered BMI, suggest the possibility of substance misuse. The question demands the most appropriate next step in managing her condition. PLANNING: This situation involves a balance between effectively managing the patient's chronic pain condition and addressing her potential substance misuse. The assumptions here could be: (i) the patient needs a stronger opioid or a different pain medication to manage her pain; (ii) her symptoms of lethargy, slow and slurred speech, and pinpoint pupils suggest opioid misuse; or (iii) her unusual drug-seeking behaviors (e.g., asking for more potent opioid or a change in prescription, taking morphine without prescription) indicate an addiction problem. While chronic pain management is important, it is crucial to clinically prioritize questioning the assumption that she might have developed substance misuse or addiction. CLINICAL REASONING: Chronic pelvic pain, particularly following invasive procedures like a hysterectomy, is not uncommon and can be debilitating for the patient, affecting their quality of life. However, it's unusual that the pain remains severe and unaffected for three consecutive years despite taking strong opioids such as oxycodone. To understand the cause of her pain worsening during intercourse (dyspareunia) and its relationship with the continued pain after hysterectomy, exploring a diagnosis of pelvic floor dysfunction or pelvic inflammatory disease can be considered. Her symptoms such as lethargy, slow and slurred speech, and pinpoint pupils that react sluggishly all hint towards the central nervous system's depression, which could be due to opioid overuse or misuse. Further, her behaviors like requesting an increased dose of oxycodone, switching to controlled-release morphine without a doctor's consultation, combined with her low BMI and her regular smoking habit could increase the risk of substance misuse or addiction. CONCLUDING: Evaluating the patient for substance misuse becomes crucial, as it could pose significant health risks and make pain management more complex. Hence, it is necessary to identify and address any issues related to opioids first before modifying her chronic pain management plan. RESPONSE: Among the given options, the most appropriate next step in her management would be (C) to order a urine toxicology screening. This will help determine the presence of opioids in her system and validate the signs of potential substance misuse or addiction. Once this issue is addressed, the healthcare provider can revisit her pain management regimen, possibly incorporating non-opioid options. Further management would also require engaging a multidisciplinary team, including pain management specialists and addiction experts, and potentially involve a combination of non-opioid medications, physical therapy, counseling, and rehabilitation services.

**Facilitator** The consensus statement synthesizes the responses of AI_A, AI_B, AI_C, AI_D, and AI_E as follows: The question presents a 35-year-old woman suffering from chronic pelvic pain following a total abdominal hysterectomy and radiation therapy for cervical cancer. Despite taking oxycodone for three years, she reports that the medication only partially relieves her pain. She requests an increased dose or a change to controlled-release morphine, which she obtained without a prescription. Her physical symptoms, including lethargy, slow speech, and pinpoint pupils, suggest potential opioid misuse. The situation requires a balance between managing the patient's chronic pain and addressing potential substance misuse. While it's possible that the patient needs a stronger opioid or a different pain medication, her symptoms and behaviors indicate a potential addiction problem. Chronic pelvic pain following invasive procedures is not uncommon, but it's unusual that the pain remains severe despite taking strong opioids. Her symptoms hint towards the central nervous system's depression, which could be due to opioid overuse or misuse. Given these considerations, it's crucial to evaluate the patient for substance misuse, as it could pose significant health risks and complicate pain management. Therefore, the most appropriate next step in managing her condition would be to order a urine toxicology screening. This will help determine the presence of opioids in her system and validate the signs of potential substance misuse or addiction. Once this issue is addressed, the healthcare provider can revisit her pain management regimen, possibly incorporating non-opioid options. Further management would also require engaging a multidisciplinary team, including pain management specialists and addiction experts.
